# Redefining feed efficiency through the livestock gut microbiome

**DOI:** 10.3389/fphys.2026.1852759

**Published:** 2026-07-08

**Authors:** Matthew Chidozie Ogwu, Sylvester Chibueze Izah, Esther Ugo Alum, Olugbemiga Ojo Aliu, Morufu Olalekan Raimi, Ayebapreye Kari

**Affiliations:** 1 Goodnight Family Department of Sustainable Development, Appalachian State University, Boone, NC, United States; 2 Department of Community Medicine, Faculty of Clinical Sciences, Bayelsa Medical University, Yenagoa, Bayelsa, Nigeria; 3 Department of Research and Publications, Kampala International University, Kampala, Uganda; 4 Department of Environmental Biology, Auchi Polytechnic, Auchi, Edo, Nigeria; 5 Niger-Delta Institute for Emerging and Re-emerging Infectious Diseases, Federal University Otuoke, Otuoke, Bayelsa, Nigeria; 6 Department of Microbiology, Faculty of Science, Bayelsa Medical University, Yenagoa, Bayelsa, Nigeria

**Keywords:** feed efficiency, gut microbiome, livestock production, nutrient bioavailability, rumen microbiome, short-chain fatty acids, sustainable animal production

## Abstract

Feed efficiency remains a central goal in livestock production because it determines both economic viability and environmental performance. Yet conventional measures such as feed conversion ratio and residual feed intake often treat efficiency as a host-level outcome and do not fully capture the biological processes that govern nutrient transformation and use. This perspective argues that the gastrointestinal microbiome is a critical, and still underappreciated, mediator of feed efficiency across livestock systems. In ruminants, rumen microbial communities drive the conversion of fibrous feeds into volatile fatty acids and microbial protein, thereby shaping host energy supply, nitrogen utilization, and methane loss. In monogastrics, intestinal microbiota influence nutrient salvage, short-chain fatty acid production, barrier integrity, immune tone, and metabolic signaling, with direct consequences for growth and productive performance. We contend that feed efficiency should be reframed as an emergent property of diet–microbiome–host interactions rather than as a simple input–output trait. From this viewpoint, microbial mediation helps explain between-animal variation in nutrient bioavailability, digestive stability, inflammatory burden, and resilience under commercial production conditions. We further highlight how microbiome-informed feeding strategies, including dietary bioactives, probiotics, prebiotics, enzymes, and precision nutrition approaches, could improve nutrient conversion while reducing methane emissions and reliance on antibiotics. Recognizing the microbiome as a functional regulator of feed efficiency offers a more mechanistic and sustainability-oriented framework for livestock nutrition research and practice, with important implications for breeding, management, and future multi-omics innovation.

## 1 Introduction

Livestock systems are under increasing pressure to produce more animal protein with fewer inputs and lower environmental costs. Rising demand for meat, milk, and eggs is occurring alongside intensifying concerns over feed costs, antimicrobial use, greenhouse gas emissions, and the long-term resilience of animal production systems. Because feed commonly represents the largest single cost in livestock production, improving feed efficiency remains one of the most economically important and environmentally consequential goals in animal agriculture (Connor, 2015; Kenny et al., 2018; Cantalapiedra-Hijar et al., 2018). Sustainable livestock production increasingly requires integrated approaches that simultaneously optimize productivity, nutrient cycling, environmental performance, and resource-use efficiency (Ogwu et al., 2025a).Feed efficiency has traditionally been evaluated using feed conversion ratio (FCR) and residual feed intake (RFI). These metrics remain valuable phenotypes, but they describe the net result of multiple processes without representing microbial metabolic pathways, host-microbe competition for substrates, inflammatory maintenance costs, or energetic and nitrogen losses (Cantalapiedra-Hijar et al., 2018; Derno et al., 2019; Brito et al., 2020; O’Hara et al., 2020). Consequently, current models cannot reliably explain why animals with comparable diets and host genetics differ in fermentation products, methane output, nutrient retention, gut stability, and productive performance (Connor, 2015; Kenny et al., 2018; Delgado et al., 2019). The central unresolved problem is not whether the microbiome is associated with efficiency, but which microbial functions causally alter efficiency, how those functions can be measured reproducibly, and whether they improve prediction across breeds, diets, life stages, and commercial environments.

A growing body of evidence identifies the gastrointestinal microbiome as a mediator rather than a passive correlate of animal performance (Maki et al., 2019; Jha et al., 2019). The causal sequence considered in this review is: diet supplies substrates; microbial enzymes and fermentation pathways transform those substrates; microbial products such as volatile or short-chain fatty acids, microbial protein, ammonia, bile-acid derivatives, and methane alter the quantity and form of nutrients available; and those products modify host epithelial, metabolic, endocrine, and immune responses (Stergiadis et al., 2021; Teklebrhan and Tan, 2022). The combined effects are expressed as measurable variation in growth, milk or egg output, FCR, RFI, nitrogen retention, disease resilience, and emissions (O’Hara et al., 2020; Liu et al., 2021). The shared principle across livestock is microbial conversion of otherwise inaccessible substrates into metabolites that change nutrient availability and host physiology, but the dominant mechanisms are not interchangeable (Lindsay et al., 2020; Cordeiro et al., 2022). In ruminants, foregut fermentation precedes host digestion and supplies most fermentative energy while also creating substantial methane and ammonia losses; microbial protein then becomes a major amino-acid source (Li and Guan, 2017; O’Hara et al., 2020; Hendawy et al., 2021). In pigs and poultry, host enzymatic digestion dominates the small intestine, whereas distal-gut fermentation contributes a smaller energy fraction and influences efficiency mainly through fiber salvage, epithelial integrity, immune activation, pathogen exclusion, and signaling (Jha et al., 2019; Tan et al., 2021). Accordingly, a microbial marker or intervention should not be generalized across these digestive systems without species-specific validation.

This perspective contributes a function-first, cross-species synthesis that links microbial substrate use to metabolite production, host response, and measurable efficiency outcomes. Rather than proposing a universal taxonomic signature, it organizes evidence into a diet-microbiome-host framework that distinguishes shared principles from digestive-system-specific mechanisms and identifies the measurements required to move from association to prediction. This framework is intended to complement FCR and RFI with mechanistic indicators that can guide precision feeding, breeding, emissions mitigation, and field validation.

## 2 Rethinking feed efficiency: beyond nutrient input–output models

Conventional feed-efficiency frameworks have enabled major gains in breeding and nutrition, but FCR and RFI remain phenomenological endpoints (Cantalapiedra-Hijar et al., 2018; McLoughlin et al., 2020). They do not directly represent microbial gene expression, fermentation stoichiometry, host-microbe competition for amino acids and energy, epithelial and immune maintenance costs, or energy and nitrogen lost as methane, heat, ammonia, and excreta (Li and Guan, 2017; Cantalapiedra-Hijar et al., 2018; Andrade et al., 2022). Two animals can therefore have similar intake and output at one time point while differing in the biological routes that produced those outcomes, their resilience to perturbation, and the environmental cost of production (Connor, 2015; Kenny et al., 2018).

Feed composition is not equivalent to nutrients delivered to host tissues. In cattle, microbial hydrolysis and fermentation of fiber generate volatile fatty acids; greater propionate capture can support gluconeogenesis and productive energy use, whereas hydrogen flow to methanogenesis removes dietary energy and increases emissions (Li and Guan, 2017; Alabi et al., 2024; Hendawy et al., 2021). Microbial degradation of protein and urea recycling determine whether nitrogen becomes microbial protein for growth or milk or is lost as ammonia and urinary nitrogen (Rosmalia et al., 2022; Suh et al., 2022). In pigs and poultry, microbial fermentation of resistant carbohydrates produces SCFAs that support epithelial function and immune homeostasis, potentially reducing nutrients diverted to inflammation, but excessive fermentation or dysbiosis can impair digestibility and performance (Jha et al., 2019; Tan et al., 2021; Solís-Cruz et al., 2020). These pathways connect microbial activity to average daily gain, milk yield, FCR/RFI, nitrogen-use efficiency, morbidity, and methane intensity rather than to nutrient availability alone (Matthews et al., 2019; Wang et al., 2020; Jha et al., 2019).

A further limitation is that digestive variation is commonly absorbed into residual error instead of modeled as a biological source of prediction. Metatranscriptomic and metagenomic studies have linked microbial genes and active pathways to FCR, RFI, growth, and intake, demonstrating that functional profiles can add information beyond taxonomy (Li and Guan, 2017; Lima et al., 2019). However, reported prediction accuracies are often based on small, system-specific cohorts, and external validation across diets, breeds, ages, sampling sites, and analytical pipelines remains limited (Delgado et al., 2019). Current models therefore fail not because microbial information is irrelevant, but because standardized functional features, causal intermediates, and sufficiently large reference populations are still lacking (Delgado et al., 2019; Paz et al., 2018; Myer et al., 2015).

Cross-species synthesis requires separating common architecture from different physiological leverage points (Tardiolo et al., 2025). Both ruminants and monogastrics exhibit the sequence diet to microbial function to metabolite to host response to performance. In ruminants, the largest leverage points are foregut fiber conversion, VFA proportions, microbial protein synthesis, and methane and ammonia losses (Hassan et al., 2020; Ge et al., 2023; O’Hara et al., 2020). In pigs and poultry, the principal leverage points are distal fermentation, barrier integrity, immune cost, bile-acid transformation, and pathogen resistance after most digestible nutrients have already encountered host enzymes (Jha et al., 2019; Ban and Guan, 2021; Atuahene et al., 2025). Thus, shared functional categories can organize comparison, but effect sizes, biomarkers, and interventions must remain species and production-system specific. Systems-based approaches are increasingly recognized as necessary for addressing interconnected sustainability challenges across food and agricultural systems (Ogwu, 2025).

**Figure 1:**
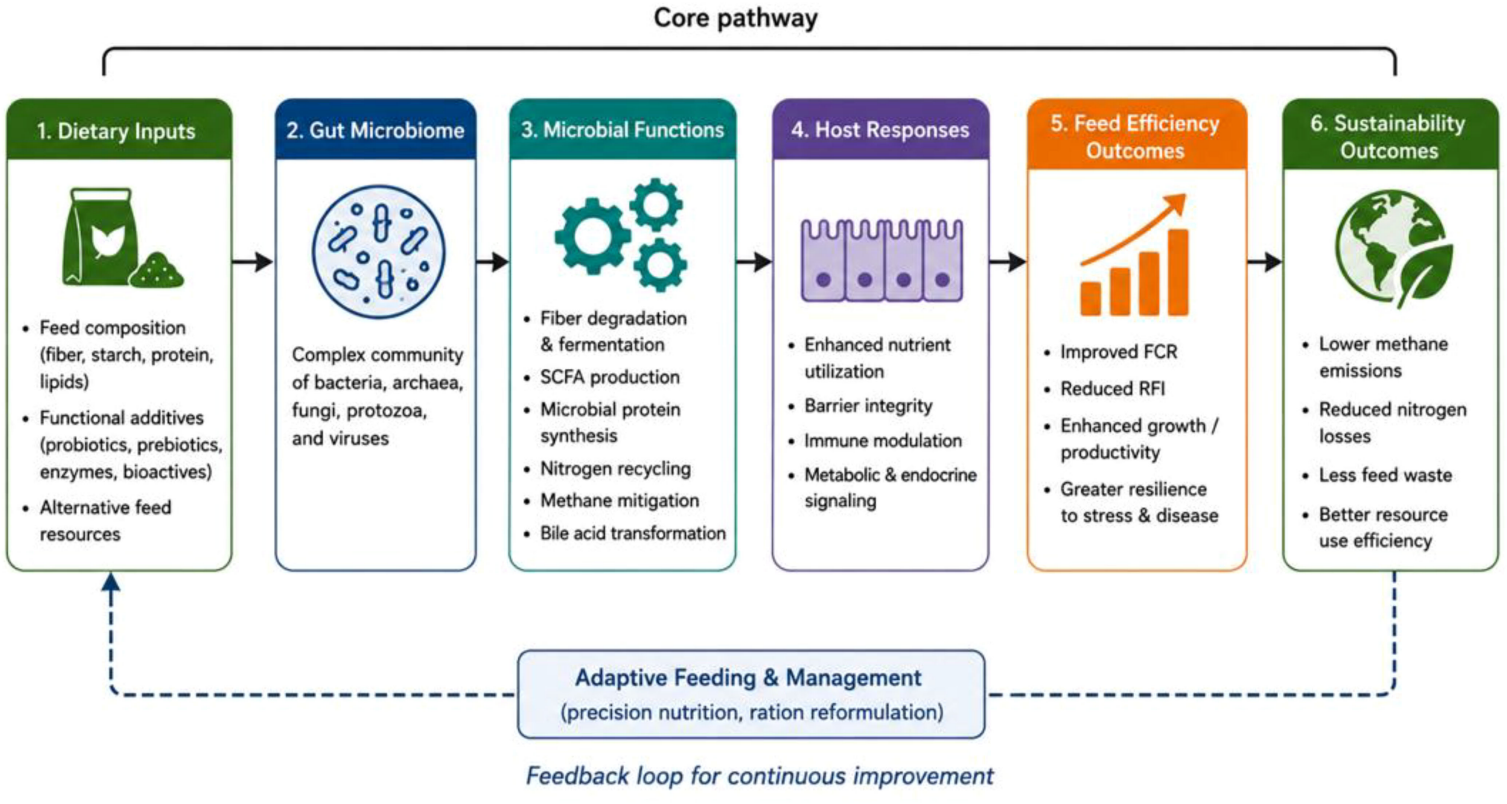
Conceptual framework illustrating microbiome-mediated feed efficiency in livestock production systems.

The diet-microbiome-host axis is used here as an underutilized analytical framework rather than a general metaphor. Existing reviews have separately examined cattle production and health, ruminal nutrient metabolism, poultry microbiota, feed-additive alternatives, persistent cattle microbiome manipulation, and diet-health interactions (Clemmons et al., 2019; Gadde et al., 2017; Liu et al., 2021; Maki et al., 2019; O’Hara et al., 2020; Williams et al., 2021). Their collective gaps are limited cross-species mechanistic alignment, inconsistent linkage of microbial functions to standardized efficiency outcomes, and insufficient guidance on how microbial data should enter predictive models (Li and Guan, 2017; Delgado et al., 2019; Xue et al., 2022). The perspectives presented here attempt to address those gaps by mapping each mechanism to a measurable mediator and outcome, separating ruminant and monogastric pathways, and specifying validation requirements for microbiome-informed prediction and intervention. **Figure 1** conceptualizes feed efficiency as an emergent property of diet–microbiome–host interactions. Dietary inputs shape the composition and functional activity of the gut microbiome, which mediates nutrient transformation through key microbial processes including fermentation, short-chain fatty acid production, microbial protein synthesis, nitrogen recycling, and methane regulation. These microbial functions influence host physiological responses, including nutrient utilization, intestinal integrity, immune regulation, and metabolic signaling, ultimately affecting feed efficiency outcomes such as feed conversion ratio (FCR), residual feed intake (RFI), productivity, and resilience (Cantalapiedra-Hijar et al., 2018; Kogut, 2019; Atuahene et al., 2025). Improved feed efficiency contributes to broader sustainability outcomes, including reduced methane emissions, lower nutrient losses, decreased feed waste, and enhanced resource-use efficiency (Cantalapiedra-Hijar et al., 2018; Atuahene et al., 2025). Feedback from production and sustainability outcomes informs adaptive feeding and management strategies, creating a continuous improvement cycle for sustainable livestock production (**Figure 1**).

## 3 The gastrointestinal microbiome as a metabolic engine

Calling the gastrointestinal microbiome a metabolic engine is operationally useful only if the engine can be measured. Here, microbial engine performance comprises five linked quantities: the range and rate of substrates degraded; the yield and profile of useful products such as VFAs, SCFAs, and microbial protein; the fraction of energy or nitrogen lost through methane, ammonia, or inefficient end products; the stability of these functions during dietary or environmental perturbation; and the strength of association with host outcomes such as FCR, RFI, growth, milk yield, egg output, and emissions (Li and Guan, 2017; Xue et al., 2022; Cantalapiedra-Hijar et al., 2018). Taxonomic abundance alone is therefore insufficient unless it predicts one or more of these functional quantities (Delgado et al., 2019; Xue et al., 2022).

The engine begins with substrate access. Microbial carbohydrate-active enzymes release fermentable intermediates from cellulose, hemicellulose, and resistant carbohydrates (Rabapane et al., 2022; Andersen et al., 2023). In ruminants, fermentation then partitions carbon among acetate, propionate, butyrate, microbial biomass, carbon dioxide, and methane; these competing routes determine usable energy, gluconeogenic supply, and energetic loss (López-Hernández et al., 2023; Hassan et al., 2020; Li and Guan, 2017). At the same time, peptide degradation, ammonia assimilation, urea recycling, and microbial growth determine whether dietary nitrogen is converted into microbial protein or lost (Rosmalia et al., 2022; Suh et al., 2022). In monogastrics, the same logic occurs primarily in the distal gut, but the productive effect is mediated less by total energy supply and more by fiber salvage, epithelial fuel, barrier function, immune tone, and pathogen ecology (Jha et al., 2019; Tan et al., 2021). Carbohydrate, SCFA, and nitrogen metabolism are therefore coupled components of a single conversion system rather than independent functions (Jha et al., 2019; Xue et al., 2022).

Fermentation products connect microbial conversion to host physiology. VFAs and SCFAs are absorbed as energy substrates and also influence epithelial turnover, receptor-mediated signaling, inflammatory regulation, and barrier integrity (van der Hee and Wells, 2021; Liu et al., 2024; Iwasaki et al., 2019). The production outcome depends on location and context: ruminal VFAs are quantitatively central to ruminant energy metabolism, whereas hindgut SCFAs in pigs and poultry contribute a smaller share of total energy but can alter nutrient absorption and the metabolic cost of maintaining gut and immune homeostasis (Li and Guan, 2017; Jha et al., 2019; Ma et al., 2022). These effects should be tested through paired measurements of fermentation products, host responses, and performance rather than inferred from microbial composition alone (Jha et al., 2019; Maki et al., 2019).

Nitrogen metabolism is integrated with carbon and energy flow. In ruminants, sufficient fermentable energy permits microbes to capture ammonia into biomass, which later supplies amino acids to the host; poor synchronization increases ammonia absorption and excretory nitrogen loss (Salami et al., 2021; Rosmalia et al., 2022; Uwineza et al., 2024). In poultry and swine, microbial amino-acid transformations can either recover nitrogenous substrates or generate metabolites that impose detoxification and inflammatory costs (Liao et al., 2024; Su et al., 2018; Vasquez et al., 2022). Thus, microbial protein yield, ruminal ammonia, blood or milk urea nitrogen, nitrogen retention, and excreta nitrogen are practical outcome-linked indicators of engine efficiency (Calomeni et al., 2015; Salami et al., 2021; Rosmalia et al., 2022). Microbiome plasticity provides a plausible source of between-animal variation because animals exposed to the same ration can differ in active pathways, fermentation products, and resilience. Functional differences have been observed between efficient and inefficient cattle, and microbial genes have predicted performance traits within study populations (Li and Guan, 2017; Lima et al., 2019). Nevertheless, host intake, genetics, health, and environment also shape the microbiome; association cannot by itself establish direction of causality (O’Hara et al., 2020). Longitudinal sampling, perturbation studies, and mediation analyses are needed to show that a microbial change precedes and explains a reproducible change in performance (Corander et al., 2022).

## 4 Integrated mechanisms of microbial mediation of feed efficiency

Diet and host conditions determine substrate availability; microbial pathways partition carbon and nitrogen into metabolites, biomass, and losses; metabolites and microbial structures alter epithelial, immune, endocrine, and intake responses; and community resilience determines whether these functions persist under stress (Kaur et al., 2023). The system is evaluated by linking each layer to a measurable endpoint: digestibility and metabolite profiles, host-response markers, FCR or RFI, product yield, nitrogen loss, methane output, and health stability (Matthews et al., 2018). **Table 1** presents a pathway-based synthesis of how gastrointestinal microbial functions influence livestock feed efficiency. It connects microbial processes with host physiological effects, production outcomes, and sustainability implications, showing that feed efficiency emerges from diet–microbiome–host interactions rather than from feed intake and animal output alone.

**Table 1:** Microbiome-mediated pathways linking microbial functions to host responses, feed efficiency, and sustainability outcomes in livestock production systems.

Microbial mechanism	Representative microbial process	Primary host effect	Feed efficiency implication	Sustainability implication	Reference
Fiber degradation and fermentation	Breakdown of cellulose, hemicellulose, non-starch polysaccharides, and resistant carbohydrates	Increased release of fermentable substrates and usable energy	Improved nutrient extraction from fibrous and low-value feeds	Better use of low-value or fibrous feed resources	Jha et al. (2019); Liu et al. (2021); Matthews et al. (2019); Singh and Kim (2021)
SCFA/VFA production	Generation of acetate, propionate, butyrate, and related fermentation products	Energy supply, epithelial support, metabolic signaling, and gut homeostasis	Greater energy harvest and improved gut function	Reduced feed wastage through better biological conversion	Corrêa et al. (2016); Jha et al. (2019); Maki et al. (2019); Matthews et al. (2019)
Microbial protein synthesis	Conversion of nitrogenous compounds into microbial biomass	Enhanced amino acid supply to the host, especially in ruminants	Improved protein-use efficiency and productive output	Lower nitrogen losses through more efficient nitrogen capture	Cantalapiedra-Hijar et al. (2018); Liu et al. (2021); Matthews et al. (2019); O’Hara et al. (2020)
Nitrogen recycling	Utilization of ammonia, urea, peptides, and other nitrogenous substrates by microbes	Improved nitrogen retention and reduced inefficiency	Better nutrient partitioning and reduced protein wastage	Reduced ammonia and nitrogen emissions	Cantalapiedra-Hijar et al. (2018); Liu et al. (2021); Matthews et al. (2019); Singh and Kim (2021)
Methane-associated fermentation pathways	Hydrogen flow toward methanogenesis or alternative fermentation pathways	Alters the proportion of feed energy lost as methane	Greater retention of dietary energy when methane is reduced	Lower enteric methane emissions and greenhouse gas intensity	Kú-Vera et al. (2020); Matthews et al. (2019); Min et al. (2020); O’Hara et al. (2020)
Bile acid and lipid metabolism	Microbial transformation of bile acids and lipid-associated compounds	Modified lipid digestion, absorption, and metabolic signaling	Alters productive efficiency, especially in monogastrics	Potential reduction in inefficient nutrient loss	Maki et al. (2019); Wang et al. (2020); Chakaroun et al. (2020)
Barrier integrity and immune modulation	Microbial regulation of mucosal defense, epithelial integrity, and inflammatory tone	Reduced gut dysfunction and lower metabolic cost of inflammation	More nutrients available for growth and production rather than immune maintenance	Lower dependence on antibiotic-based performance support	Gadde et al. (2017); Maki et al. (2019); O’Hara et al. (2020); Williams et al. (2021)
Digestive stability and resilience	Microbial buffering of dietary changes, heat stress, pathogen pressure, and production stressors	Maintained gut functionality under changing or adverse conditions	More stable feed conversion across commercial production conditions	Greater resilience in livestock production systems	Clemmons et al. (2019); O’Hara et al. (2020); Williams et al. (2021)
Neuroendocrine and gut–brain signaling	Microbial metabolites and immune mediators affecting intake, stress response, appetite, and metabolism	Influences appetite, stress response, and physiological regulation	Contributes to between-animal variation in feed efficiency	Supports precision nutrition and management, but evidence remains more emerging than established in livestock	Wang et al. (2020); O’Hara et al. (2020); Chakaroun et al. (2020)

### 4.1 Microbial enhancement of nutrient bioavailability

Microbial communities enhance nutrient bioavailability by enzymatically transforming complex feed components into forms the host can absorb or use more efficiently (Velázquez-De Lucio et al., 2021). In ruminants, this includes the breakdown of lignocellulosic plant matter and subsequent fermentation into metabolically useful end products (Li, 2021). In monogastrics, microbial activity can improve access to otherwise resistant carbohydrates, modulate mineral liberation, and alter the digestion of lipids and proteins (Sureshkumar et al., 2023). Microbially derived enzymes used in feed, including phytases, carbohydrates, and proteases, have long been recognized as tools for improving growth and feed efficiency, particularly in poultry and swine (Gadde et al., 2017). The broader implication is that nutrient bioavailability is a co-produced outcome of feed chemistry and microbial metabolism. Improved nutrient utilization is central to sustainable resource management and reducing inefficiencies across biological production systems (Ogwu and Kosoe, 2025a).

Microbial activity also affects the release of bound micronutrients and the transformation of compounds that influence absorption. For example, microbial bile salt hydrolase activity in poultry can alter bile acid pools and, in turn, affect lipid and vitamin absorption, demonstrating that microbial metabolism can either enhance or constrain productive efficiency depending on community composition and functional balance (Maki et al., 2019). Such findings suggest that the most relevant nutritional unit is not the diet as formulated, but the diet as metabolically processed by the microbiome. Within the integrated framework, bioavailability is the entry layer rather than the final outcome. Its contribution to efficiency must be confirmed by downstream changes in absorbed metabolites, tissue or product deposition, and reduced nutrient loss.

### 4.2 Microbial metabolites and host energy harvest

Microbial metabolites are central to host energy harvest. In ruminants, fermentation products generated by the rumen microbiota provide the biochemical foundation for converting forages into animal products (van der Hee and Wells, 2021; Lv et al., 2021). In monogastrics, SCFAs produced in the hindgut contribute to local epithelial health and systemic metabolic regulation (Hu et al., 2023). These metabolites can influence satiety, epithelial turnover, inflammatory tone, and nutrient partitioning, reinforcing the idea that microbial fermentation shapes both the quantity and quality of energy available to the host (Corrêa et al., 2016; Abdul Rahim et al., 2019). Improving biological conversion efficiency is increasingly recognized as a pathway toward reducing waste generation and enhancing circularity within production systems (Ogwu and Kosoe, 2025b).

The efficiency implications are substantial. When microbial fermentation channels hydrogen and carbon into energetically favorable pathways such as propionate production rather than methane, more feed energy can be retained within the productive system (Tseten et al., 2022). Reviews of rumen microbiome function and methane mitigation highlight that shifting fermentation away from methanogenesis can improve metabolizable energy use while reducing environmental burden (Matthews et al., 2019; Kú-Vera et al., 2020). Thus, feed efficiency should not be framed only as intake relative to output, but also as the extent to which microbial metabolism minimizes energetic leakage (Kú-Vera et al., 2020). This energy-harvest layer feeds directly into signaling and partitioning: the same metabolite profile that changes retained energy can also alter epithelial and endocrine responses, so production outcomes should be interpreted together with methane and host metabolic indicators (Krištolaitytė et al., 2025).

### 4.3 Microbiome–host signaling pathways

The microbiome also shapes feed efficiency through host signaling pathways. Gut microbes influence the gut–immune axis by regulating epithelial development, mucosal defense, inflammatory tone, and immune maturation. Since chronic low-grade inflammation diverts nutrients away from growth and production toward immune activity, microbial communities that support intestinal homeostasis may improve efficiency indirectly by reducing this metabolic burden (O’Hara et al., 2020; Maki et al., 2019).

The microbiome–gut–brain axis adds another layer. Microbial metabolites and immune mediators can affect feed intake behavior, stress responses, and neuroendocrine signaling. In swine, bidirectional crosstalk between gut microbiota and host metabolism is increasingly recognized, including through pathways that connect intestinal microbial activity with broader physiological regulation (Wang et al., 2020). While this area remains less resolved in production settings than in biomedical models, it is plausible that microbial mediation of appetite, stress, and inflammatory status contributes meaningfully to between-animal variation in feed efficiency (Wessels, 2022). Because evidence for neuroendocrine mediation is less mature in commercial livestock, this pathway should be treated as a testable component of the system, not as an established explanation, and evaluated with intake behavior, stress, immune, and performance data (Gouvêa et al., 2022).

### 4.4 Microbial regulation of digestive stability

Microbial mediation also involves stabilization. A resilient microbiome can buffer dietary variability, resist pathogen overgrowth, and maintain fermentation performance under nutritional or environmental stress (Kaur et al., 2023). This stabilizing function matters because inconsistent digestion and gut dysfunction often reduce productive efficiency even when diets appear adequate on paper. Dietary interventions that support microbial resilience have therefore been proposed as sustainable tools to maintain health and productivity in reduced-antimicrobial systems (Williams et al., 2021).

Digestive stability is especially relevant under suboptimal feeding conditions, heat stress, pathogen challenge, or abrupt ration changes (Rodrigues et al., 2022). In such contexts, a functionally robust microbiome may preserve nutrient extraction and barrier integrity better than a dysbiotic community. This suggests that microbial resilience should be considered part of feed efficiency, particularly in commercial systems where animals routinely encounter fluctuating conditions (St-Pierre et al., 2023; Kaur et al., 2023). Resilience closes the framework by determining the repeatability of bioavailability, metabolite, and signaling effects over time. A candidate microbial signature is operationally valuable only if it remains predictive during diet changes, heat, infection, and other commercial perturbations (Mârza et al., 2025).

## 5 Diet–microbiota–efficiency interactions

Diet is one of the strongest drivers of gut microbial composition and function, making the diet–microbiota relationship a practical entry point for improving feed efficiency (Tardiolo et al., 2025). Feed ingredients do not simply nourish the host; they select for microbial guilds, alter fermentation pathways, and change the balance of metabolites that shape host physiology (Wang et al., 2021). In ruminants, ration chemistry strongly influences rumen fermentation patterns and the abundance of functional groups linked to fiber degradation, propionate formation, and methanogenesis (Liu et al., 2021; Matthews et al., 2019). In poultry and swine, early-life diet and additive exposure can affect microbial succession, community stability, and metabolite production with lasting effects on gut development and performance (Maki et al., 2019; Wang et al., 2020).

Plant-derived bioactives offer one especially promising route for microbiome-mediated intervention. Tannins, saponins, essential oils, and other secondary metabolites can alter microbial populations and fermentation pathways, sometimes reducing methane production or shifting energy flow toward more efficient products such as propionate (Kú-Vera et al., 2020; Min et al., 2020). However, these interventions are not universally beneficial. Their effects depend on dose, diet background, animal species, and the structure of the resident microbiome (Adhikari and Kim, 2017; Cunningham et al., 2021). This variability reinforces the need for microbiome-informed feeding strategies rather than one-size-fits-all supplementation.

Prebiotics and probiotics have also attracted attention as alternatives to antibiotic growth promoters. In poultry, reviews indicate that probiotics and other microbiota-modulating interventions can improve feed efficiency, although outcomes remain context dependent (Gadde et al., 2017; Roto et al., 2015). Fermented feeds, synbiotics, microbial enzymes, and alternative protein sources likewise influence microbial ecology and nutrient use (Olukomaiya et al., 2019). Together, these interventions point to a dynamic feedback system in which diet shapes microbes, microbes shape nutrient fate, and nutrient fate shapes production efficiency.

This feedback loop should be central to future livestock nutrition. Rather than designing diets solely around proximate composition, nutritionists and breeders may need to formulate for desired microbial functions, such as fiber degradation efficiency, SCFA production, reduced methanogenesis, nitrogen retention, or improved barrier integrity. These strategies should therefore be evaluated by whether they reproducibly increase SCFA production, reduce methanogenesis, improve nitrogen retention, or strengthen barrier integrity (Atuahene et al., 2025). Doing so would move feeding strategies from static ration balancing toward functional ecosystem management within the gut (Wang et al., 2021). Evidence across interventions is not uniformly consistent. Additives that shift fermentation *in vitro* may have smaller, transient, or diet-dependent effects *in vivo* (FAO, 2020). A taxon associated with efficiency in one cohort may be absent or non-predictive in another because feed-efficiency microbiomes are shaped by diet, genotype, age, sex, and production context (McLoughlin et al., 2020). An intervention that improves gut health may not improve FCR when baseline health is already high or when animals are not under a relevant challenge (Atuahene et al., 2025). The more defensible synthesis is functional: successful strategies should reproducibly shift a defined pathway or metabolite, produce the expected host response, and improve a pre-specified production or environmental endpoint (Zou et al., 2022).

## 6 Microbial signatures of high feed efficiency

A growing number of studies suggest that more efficient animals harbor distinct microbial configurations relative to their less efficient counterparts (Myer et al., 2015; Li and Guan, 2017; McLoughlin et al., 2020). In cattle, associations have been reported between feed efficiency and the composition of the rumen microbiome, including taxa linked to fermentation balance, hydrogen use, and methane output (O’Hara et al., 2020; Matthews et al., 2019). Some reviews note that higher feed efficiency has been associated with lower abundance of methanogenic archaea and microbial profiles that favor more productive energy capture, although findings are not always consistent across breeds, diets, and analytical pipelines (Kú-Vera et al., 2020).

This inconsistency should not be interpreted as failure; instead, it suggests that microbial signatures of efficiency may be more functional than purely taxonomic (Xue et al., 2022). Diversity indices alone may be insufficient because feed-efficiency differences can occur through functional gene abundance, gene expression, fermentation profiles, and metabolomic outputs rather than simple presence–absence patterns of particular genera (Li and Guan, 2017). A microbiome associated with high efficiency in one production system may not be taxonomically identical to that in another, but the underlying functions, such as enhanced polysaccharide breakdown, lower methane loss, stronger barrier homeostasis, or improved nitrogen capture, may converge (Andrade et al., 2022). This has important implications for breeding and selection. Host genetics undoubtedly shapes feed efficiency, but microbial traits may offer complementary biomarkers or even selectable phenotypes (Brito et al., 2020). The challenge is to identify microbial features that are robust, predictive, and transferable enough for field application (Xue et al., 2022). Future selection programs may need to integrate host genotype, microbial function, and environmental context rather than treating them as separate domains (Wen et al., 2021).

Inconsistent taxonomic signatures should not be dismissed, but they limit universal biomarker claims (McLoughlin et al., 2020). Different taxa can encode redundant functions, while sampling site, diet, breed, age, and bioinformatic workflow can alter observed associations (Lopes et al., 2021). Studies have reported both distinguishable functional profiles and weak community-level separation between efficiency groups (Xue et al., 2022). Functional gene abundance, gene expression, proteins, metabolites, and fermentation phenotypes may therefore be more transferable than genus-level lists, but this hypothesis requires blinded external validation (Brito et al., 2020). A useful signature must predict outcomes in animals, farms, and conditions not used to train the model (Xue et al., 2022).

## 7 Toward microbiome-informed feeding strategies

The next phase of livestock nutrition should be microbiome-informed. Precision nutrition has traditionally focused on matching nutrient supply to host requirements, but an expanded model would also aim to match diet design to microbial functionality. This means formulating rations not only for protein, energy, and minerals, but also for fermentation outcomes, microbial stability, and metabolite profiles. Future livestock systems will likely benefit from integrated sustainability frameworks that combine biological efficiency, environmental stewardship, and technological innovation to optimize production outcomes while minimizing ecological costs (Ogwu et al., 2025b).

Several intervention paths are already visible. Synbiotic combinations may support beneficial fermentation and barrier health. Phytogenic additives may redirect fermentation or suppress inefficient pathways such as excessive methanogenesis. Enzyme technologies can enhance substrate accessibility and change downstream microbial metabolism. Persistent microbiome manipulation remains challenging, especially in cattle, but growing knowledge of host–microbiome interactions suggests that targeted and context-aware interventions are feasible (Clemmons et al., 2019; Matthews et al., 2019).

A field-ready workflow would first define the target function and outcome, then screen a diet or additive for dose response and safety, verify the microbial and metabolite mechanism, and finally test performance in multi-farm trials against an economic threshold. Major constraints include sequencing and metabolomics costs, sample collection and cold-chain requirements, batch and pipeline effects, strain-specific efficacy, transient colonization, variable baseline diets and microbiomes, regulatory approval, manufacturing consistency, and the need for rapid decision turnaround. Near-term implementation is therefore more likely to use low-cost proxies such as fermentation products, methane phenotypes, fecal metabolites, or targeted microbial panels than routine whole-metagenome profiling of every animal (Clemmons et al., 2019; Delgado et al., 2019).

Digital agriculture can support this staged approach by combining targeted microbial or metabolite measurements with intake, weight, milk, egg, health, and environmental sensor data. However, machine-learning performance must be reported with independent validation, calibration drift, sampling cost, and decision value. A model that classifies animals within one research cohort is not yet a deployable feeding tool; deployment requires reproducible sampling, interpretable features, timely outputs, and a demonstrated return above the cost of measurement and intervention.

## 8 Sustainability implications of microbial feed efficiency

Microbiome-mediated improvements in feed efficiency could generate benefits that extend beyond production economics (FAO, 2020). Better nutrient conversion means less feed is required per unit of meat, milk, or eggs, reducing pressure on land, water, and input-intensive feed supply chains (Sandström et al., 2022). Microbial pathways also strongly influence methane formation and nitrogen loss, both of which are major sustainability concerns in livestock systems (Oenema and Oenema, 2022). Enteric methane represents both a greenhouse gas liability and a loss of dietary energy, estimated in ruminants at 2–12% of gross intake depending on diet and system (Tseten et al., 2022). Strategies that shift microbial metabolism away from methane and toward productive fermentation therefore offer a dual gain in efficiency and environmental performance (Kú-Vera et al., 2020).

Microbiome-informed feeding may also reduce reliance on antibiotics by improving gut resilience, suppressing pathogen expansion, and supporting mucosal immunity (Rahman et al., 2022). This is especially important as producers seek alternatives to antibiotic growth promoters and regulators intensify scrutiny of antimicrobial use in animal agriculture (Vieco-Saiz et al., 2019). Microbial feed efficiency also aligns with circular bioeconomy thinking. Livestock can convert by-products and non-human-edible biomass into valuable food, but microbiome-targeted strategies could further enhance the use of unconventional feed resources, plant residues, or bioactive-rich side streams while maintaining animal health and product quality (Sandström et al., 2022). These sustainability benefits should be quantified rather than assumed. Trials should report output-adjusted measures such as methane or nitrogen loss per kilogram of gain, milk, or eggs, together with feed inputs, product yield, health events, and intervention costs (FAO, 2020). This prevents an apparent reduction in emissions or antimicrobial use from being interpreted as improved efficiency when production is simultaneously reduced (FAO, 2020).

## 9 Challenges and knowledge gaps

The principal knowledge gap is causal and predictive integration. Current studies often identify taxonomic associations but do not measure the full chain from diet and microbial function through metabolites and host responses to FCR, RFI, product yield, or emissions (Xue et al., 2022). Small cohorts, cross-sectional designs, inconsistent sampling sites, and nonstandard pipelines limit reproducibility and causal inference (Enokela et al., 2026). Consequently, current models do not yet explain which microbial functions are necessary, sufficient, or merely correlated with high efficiency, nor whether a marker discovered in one breed or diet will remain predictive elsewhere (Lopes et al., 2021). Despite its promise, microbiome-centered feed-efficiency science faces several limitations. Microbiome composition is highly variable across species, breeds, ages, diets, geographies, and management systems (Tardiolo et al., 2025). This makes it difficult to identify universal signatures of efficiency or to translate findings directly from experimental settings to commercial operations. Taxonomic associations are often inconsistent, and functional readouts remain underdeveloped in many livestock studies (Li and Guan, 2017).

Methodological differences also complicate interpretation. Sampling location, sequencing platform, bioinformatic pipeline, and analytical scale can all influence conclusions (Enokela et al., 2026). Cross-sectional studies dominate much of the literature, limiting causal inference. Moreover, many proposed microbiome interventions show variable persistence, raising questions about how stable or durable engineered microbial states can be in the face of changing diets and environments (Zhou et al., 2018). There are also practical and regulatory barriers. Microbiome-directed additives must be cost-effective, safe, scalable, and supported by evidence of consistent efficacy (FAO, 2020). Producers need clear value propositions, not abstract promises. Regulators require evidence that benefits are reproducible across relevant production conditions. These realities mean that progress will depend not only on microbiology and animal nutrition, but also on systems economics, product development, and translational field validation.

## 10 Future directions and research priorities

Future work should prioritize longitudinal, adequately powered, multi-farm studies that measure diet, microbial function, metabolites, host physiology, and efficiency outcomes on the same animals (Xue et al., 2022). Discovery cohorts should be separated from blinded external validation cohorts, and models should be challenged across breeds, diets, life stages, climates, and management systems. Multi-omics should be used selectively to identify a minimal, reproducible feature set rather than treated as an endpoint in itself (Brito et al., 2020).

A staged predictive framework can complement FCR and RFI: diet and environment define inputs; microbial pathway activity and fermentation products define transformation; epithelial, immune, endocrine, and metabolic markers define host response; and growth, milk or egg yield, nitrogen retention, methane intensity, and health stability define outputs (Zhou et al., 2024). Candidate microbial efficiency indices should be species- and system-aware, pre-specified, repeatable over time, and shown to improve prediction beyond host genetics, diet, and conventional phenotypes (Wen et al., 2021). Cross-disciplinary collaboration is essential, but success should be judged by external predictive accuracy, mechanistic coherence, cost, and actionable improvement under commercial conditions.

## 11 Conclusion

Feed efficiency should no longer be viewed solely as a function of feed intake and animal output, but as an emergent systems trait shaped by dynamic interactions among diet, the microbiome, host physiology, and the production environment. This review advances the field by integrating evidence across ruminant and monogastric livestock into a unified mechanistic framework that links dietary substrates to microbial pathways, metabolite production, host responses, and measurable production and sustainability outcomes. In doing so, it highlights both the shared architecture of microbiome-mediated feed efficiency and the species-specific mechanisms that determine how microbial processes influence nutrient utilization, productivity, resilience, methane emissions, and nitrogen losses. Existing evidence suggests that the greatest opportunity lies not in identifying universal microbial taxa, but in understanding and validating microbial functions that consistently improve nutrient conversion and animal performance across diverse production systems. Microbiome-derived information has the potential to complement conventional metrics such as FCR and RFI by providing mechanistic insight into why animals differ in efficiency and by revealing intervention points that are invisible to traditional phenotypes. Realizing this potential will require a shift from descriptive association studies toward predictive, causally informed, and externally validated frameworks capable of performing under commercial conditions. The ultimate measure of success will not be the discovery of novel microbial signatures, but the development of practical, cost-effective strategies that reliably improve growth, milk and egg production, nutrient retention, animal health, and environmental performance. As livestock production faces increasing demands for efficiency and sustainability, the microbiome represents one of the most promising frontiers for transforming feed efficiency from a retrospective outcome into a manageable and predictable biological process.

## Data Availability

The original contributions presented in the study are included in the article/supplementary material. Further inquiries can be directed to the corresponding author/s.
